# Age-Matched, Case-Controlled Comparison of Clinical Indicators for Development of Entropion and Ectropion

**DOI:** 10.1155/2014/231487

**Published:** 2014-03-05

**Authors:** Kevin S. Michels, Craig N. Czyz, Kenneth V. Cahill, Jill A. Foster, John A. Burns, Kelly R. Everman

**Affiliations:** ^1^Division of Ophthalmology, Section Oculofacial Plastic and Reconstructive Surgery, Ohio University/Doctor's Hospital, 50 Old Village Road, Columbus, OH 43228, USA; ^2^Department of Ophthalmology, Oral and Maxillofacial Surgery, Grant Medical Center, 111 S. Grant Avenue, Columbus, OH 43215, USA

## Abstract

*Purpose.* To analyze the clinical findings associated with involutional entropion and ectropion and compare them to each other and to age-matched controls. *Methods.* Prospective, age-matched cohort study involving 30 lids with involutional entropion, 30 lids with involutional ectropion, and 52 age-matched control lids. *Results.* The statistically significant differences associated with both the entropion and ectropion groups compared to the control group were presence of a retractor dehiscence, presence of a “white line,” occurrence of orbital fat prolapse in the cul-de-sac, decreased lower lid excursion, increased lid laxity by the snapback test, and an increased lower lid distraction. Entropion also differed from the control group with an increased lid crease height and decreased lateral canthal excursion. Statistically significant differences associated with entropion compared to ectropion were presence of a retractor dehiscence, decreased lateral canthal excursion, and less laxity in the snapback test. *Conclusion.* Entropic and ectropic lids demonstrate clinically and statistically significant anatomical and functional differences from normal, age-matched lids. Many clinical findings associated with entropion are also present in ectropion. Entropion is more likely to develop with a pronounced retractor deficiency. Ectropion is more likely to develop with diminished elasticity as measured by the snapback test.

## 1. Introduction

Multiple anatomical defects are believed to contribute to involutional entropion, and numerous surgical techniques have been described to correct them. The three anatomic factors most consistently described in the literature as requiring attention are lower lid retractor disinsertion, horizontal lid laxity, and orbicularis oculi muscle override [1–11]. Horizontal lid laxity, diminished orbicularis tone, and lower lid retractor disinsertion have all been implicated in the development of involutional ectropion [12–15].

**Figure 1:**
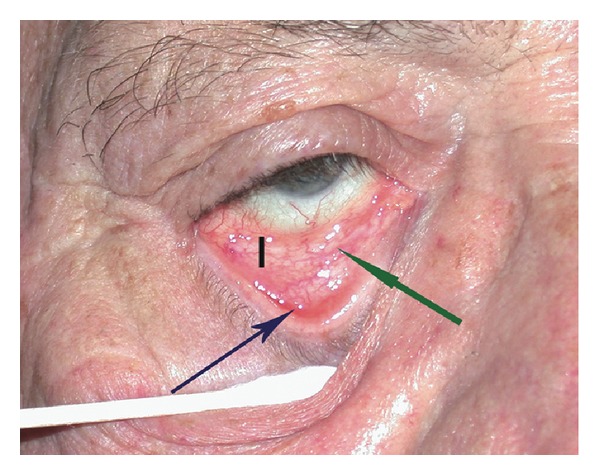
Patient with entropion of the right lower eyelid. Blue arrow demonstrates retractor dehiscence with “V” shaped junction between the retractors and the inferior border of the tarsus. Green arrow demonstrates the “white line.” Black bar indicates area of orbital fat prolapse.

**Figure 2:**
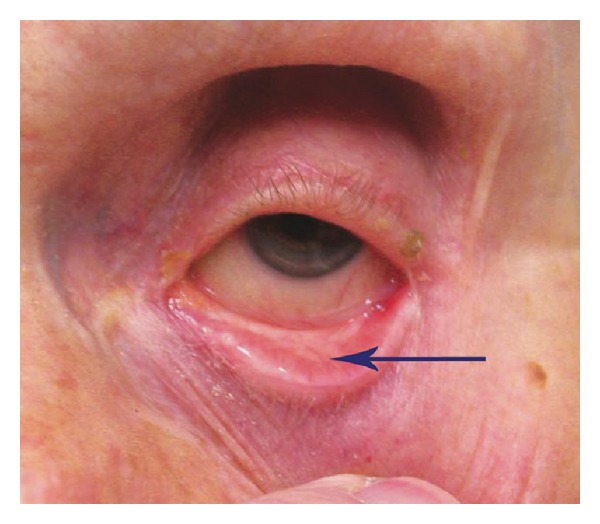
Age-matched control lower eyelid. Blue arrow points to the inferior border of the tarsus where there is not a “V” shaped junction between the retractors and inferior border of the tarsus. There is no orbital fat prolapse and no “white line.”

The anatomic and histologic features of lower eyelid malposition have been described by numerous authors. Lower lid anatomy, including the lower lid retractors, was investigated by Jones who theorized that laxity of the retractors would allow the inferior border of tarsus to rotate outward [2]. He described lower lid retractor plication and advancement as a surgical treatment for entropion [3]. Jones [2] also postulated that lower lid retractor laxity was analogous to a levator aponeurosis dehiscence. Collin and Rathbun [16] histologically studied patients with entropion versus normal eyelids evaluating the lower lid retractors. In the entropion specimens, they found that the lower lid retractors and orbital septum only came to within 3.5 mm of the inferior border of the tarsus versus 1.5 to 2.5 mm in normal lids [16]. Additionally, a larger amount of orbital fat was present in the entropion specimens compared to the normal lids indicating a retractor dehiscence [16]. The tarsal plate has been shown to invert in entropion where the lower border rotates superiorly and anteriorly 16 degrees and the upper border rotates inward 63 degrees [17]. In some patients, the junction of the inferior border of the tarsus with the lower lid retractors has an acute angulation as compared to a normal eyelid. With inferior distraction of the eyelid, an abnormal cul-de-sac develops below the inferior tarsal border forming a “V” shaped appearance (Figures 1 and 2). We believe this indicates the presence of a retractor dehiscence or disinsertion. Additionally, the presence of a “white line” representing the retracted edge of the disinserted lower lid retractors under the palpebral conjunctiva may be visible and is referred to as a complete retractor disinsertion [18].

Retractor disinsertion has also been associated with ectropion. Putterman [12] and Wesley [13] described patients where lateral tightening was insufficient to correct an ectropion. They found a retractor dehiscence when surgical exploration of the lower lid was performed. Reattachment of the lower lid retractors then led to a successful ectropion treatment. Additionally, when describing ectropion, Hawes and Dortzbach [19] commented that the lower lid retractor muscle was further from the inferior tarsal border and that there was an increased amount of adipose tissue near the tarsus and capsulopalpebral fascia junction in ectropion patients. These are findings that have also been described in entropion.

Horizontal lid laxity is also thought to be important in the development of entropion [20, 21]. As surgical treatments evolved, surgeons found that recurrence of the entropion was more likely if horizontal laxity was not corrected [22–24]. Danks and Rose [25] found addressing horizontal laxity at the time of surgery in addition to advancing the lower lid retractors and eliminating orbicularis oculi muscle override increased the success rate of surgery. They recommended that horizontal lid shortening should be performed in all cases of involutional entropion [25].

Horizontal lid laxity is also thought to play a significant role in ectropion. Lateral canthal tendon lengthening and an abnormal lid traction test (lid distraction test) were found to be statistically significant when comparing ectropion to control lids [26]. The medial canthal tendon was also found to be longer in patients with ectropion compared to the control group, but the difference was not statistically significant.

The orbicularis oculi muscle is thought to play a role in involutional entropion by the preseptal orbicularis migrating superiorly over the tarsus, perhaps because of increased connective tissue laxity [16]. In a histologic study, Sisler et al. [27] found septal and tarsal atrophy in patients with entropion. In ectropion, they found orbicularis and Riolan's muscle ischemia, atrophy, and collagen fragmentation. Orbicularis oculi atrophy was also found with light and electron micrography in specimens of lids with ectropion [15, 28].

Historically, many studies have reported features associated with entropion as subjective clinical observations. Surgical interventions were aimed at addressing each of these features [1–11, 16, 22, 23, 25]. Other studies identified clinical features and compared them to the opposite unaffected lid or against lids with ectropion [29–32]. Very few studies have been done in a comparative manner in patients with entropion versus age-matched controls [21]. Kersten et al. [32] compared patients with entropion versus age-matched controls with Hertel exophthalmometry measurements and found that no statistically significant difference existed. This study went against the belief that entropion was associated with enophthalmos [33]. Benger and Musch [21] limited their study to patients over the age of 65 and found that only patients with entropion of greater than 6-week duration had increased horizontal lid laxity compared to the age-matched controls. They found a statistically significant difference in the vertical distraction test of patients with entropion compared to their control group, but the vertical excursion from up to down gaze was not significant [21]. More recently, Beigi et al. [34] did a study measuring lower lid excursion, horizontal lower lid laxity, and orbital fat prolapse. However, they used the opposite unaffected eye as a control in patients with unilateral entropion. This study did not find a difference in horizontal laxity or lower lid vertical excursion between the lid with entropion and the nonentropic eyelid. However, orbital fat prolapse was found to be associated with involutional entropion, likely related to lower lid retractor thinning and dehiscence [28].

This study represents an attempt to synthesize the information gleaned by previous studies and develop a comprehensive protocol to assess all the potential mechanisms and related clinical findings of involutional entropion and ectropion. The study was designed to evaluate and compare the clinical eyelid parameters proposed to contribute to entropion and ectropion formation. The findings will be compared with an age-matched control group to best remove any experimental bias.

## 2. Methods

This prospective age-matched, case-control study was conducted from 2009 to 2010 with the Institutional Review Board (IRB) approval. Seventy-one consecutive patients (142 eyes) were measured for this study. The eyes were assigned to the entropion group, ectropion group, opposite lid entropion control group (entropion control), opposite lid ectropion control group (ectropion control), or the age-matched control group. None of the patients had prior eyelid surgery.

Patients were evaluated for the presence or absence of involutional entropion or ectropion. The patient was observed and if the eyelid margin was rolled in toward the eye constantly or intermittently, then involutional entropion was diagnosed. If an eyelid was rolled outward either medially or along its entire length without evidence of anterior lamellar contracture or facial paralysis, involutional ectropion was diagnosed. Patients with cicatricial changes of the eyelid were not included in the study.

The patients in each of the five groups were then evaluated for nine clinical parameters as follows.

(1) margin to reflex distance 2 (MRD_2_) measured to the nearest half millimeter with a ruler as the distance between the central corneal light reflex and the lower lid margin; (2) lower lid crease measured to the nearest half millimeter using a ruler from the lower lid margin; (3) presence of a retractor dehiscence; this was deemed present when the junction of the lower lid retractors to the tarsus had a “V” shape when the lower lid was distracted inferiorly (Figure 1); (4) presence of a retractor disinsertion with the finding of a subconjunctival “white line” in the fornix (Figure 1); (5) presence or absence of orbital fat prolapse; this was deemed present if the inferior orbital fat protruded into the fornix and anterior level of the everted tarsus when the lower eyelid was distracted inferiorly (Figure 1); (6) lower lid vertical excursion as measured to the nearest half millimeter by the movement of the central lower eyelid margin from up gaze to down gaze; (7) lateral canthal excursion as measured to the nearest half millimeter by the movement of the lateral canthal angle from up gaze to down gaze; (8) lower lid laxity and orbicularis oculi muscle tone with use of the snapback test; this was assessed by observing the time taken for the lower lid margin to return to its resting position after being pulled inferiorly; results were reported on a four-point Likert scale defined as follows: (i) normal quick return; (ii) slow return; (iii) return requires one blink; (iv) return requires more than one blink; (9) horizontal lid laxity using inferior distraction of the lid; this was recorded to the nearest half millimeter by measuring the distance between the lid margin and the globe in primary gaze while pulling the lid inferiorly.

Data was analyzed utilizing parametric and nonparametric tests within SPSS, version 16.0 (SPSS, Chicago, IL). The descriptive statistics of mean, median, range, and standard deviation were calculated for each group. The independent samples *t*-test was used to interpret scaled data. Ordinal data was analyzed utilizing the Mann-Whitney *U* test (*U*). The *Z* test statistic reported for the Mann-Whitney *U* test indicates if the two samples being compared come from the same underlying distribution at the *P* = 0.05 significance level. A *Z* score of less than 1.96 indicates that the two samples come from the same underlying distribution. Nominal data was analyzed with Fisher's exact test as dictated by the expected 2 × 2 table values. All data were reported at the 0.05 alpha level with two-tail significance.

## 3. Results

Seventy-one patients (142 eyes) were enrolled in the study. The control group consisted of 26 patients (52 eyes) with a mean age of 75 (range 56–89). There were 13 males (mean age 71, range 59–89) and 13 females (mean age 79, range 56–87) in the control group. The entropion group consisted of twenty-six consecutive patients (30 eyes), 13 male and 13 female, with unilateral (22 patients) or bilateral (4 patients) entropion. The mean overall patient age was 76 years old (range 53–85). The females had a mean age of 79 (range 70–85) and the males 72 (range 53–84). The ectropion group consisted of 19 patients (30 eyes) with a mean age of 81 (range 57–92). There were seven patients with unilateral ectropion and 12 with bilateral disease. The ectropion group consisted of 12 males (21 eyes) (mean age 79, range 57–89) and 7 females (9 eyes) (mean age 84, range 75–92). A secondary control group was created using the “normal” eyelid of patients with unilateral disease. These groups were the designated entropion opposite lid control group (entropion control) and ectropion opposite lid control group (ectropion control). One patient with unilateral ectropion had scarring of the opposite lid and was not used in the ectropion control group.  Table 1 contains a summary of the descriptive statistics for the control, entropion, ectropion, and opposite lid control groups.  Table 2 contains the descriptive statistics for each clinical measurement obtained. The statistical results of all the analyzed groups are summarized in Table 3, and a summary of the statistically significant results for all groups is shown in Table 4.

No statistically significant difference was found between any of the groups for MRD_2_ measurements. The presence of a retractor dehiscence defined as a “V” shaped insertion and the parameter of a slowed return on the snapback test were both found to be statistically significant when comparing the entropion and ectropion groups to the control group. Additionally, a statistically significant difference was found between the entropion and ectropion groups for both of these measurements. A retractor dehiscence occurred more frequently in entropion and the snapback test was slower in ectropion.

The presence of a “white line” and orbital fat prolapse in the inferior cul-de-sac were statistically significantly different and were more common in the entropion and ectropion groups compared to the control group. Lower lid excursion was decreased in the entropion and ectropion groups versus the control group. Lower lid distraction was greater and statistically significant in the ectropion and entropion groups compared to the control eyes. However, no statistically significant difference between the entropion and the ectropion groups was found in these four clinical parameters.

The lid crease height was found to be statistically significantly greater in the entropion lid group than in the control group. Those eyes which did not have a measurable lid crease were excluded from the calculation. No statistical difference was found between the ectropion lid group and the control group or between the entropion and ectropion groups.

Lateral canthal excursion was diminished and was statistically significant in the entropion group as compared to both the control and the ectropion groups. The difference was not found to be statistically significant between the ectropion group and the control group.

## 4. Discussion

In unilaterally affected entropion and ectropion patients, the risk for developing a malposition in the “unaffected” lid is demonstrated by significant abnormalities when compared to the age-matched control group. While many studies have used the contralateral lid as a control, the contralateral lid in unilaterally affected patients is not a valid “normal” control because of these abnormalities. This is supported by the fact that MRD_2_ was the only variable that showed no statistical difference between entropic or ectropic lids and the control lids.

Retractor dehiscence, presence of a “white line,” orbital fat prolapse, decreased lower lid excursion, increased lower lid laxity, and increased lower lid distraction are findings associated with both entropion and ectropion. The presence of these features may promote the development of either entropion or ectropion in lids currently not exhibiting clinical changes.

Eyelids with ectropion have decreased lid elasticity compared to the entropion and control groups as demonstrated with the snapback test. An increased lid distraction test is also found in the ectropion group when compared to the age-matched control group. Alterations in the tarsus or ligamentous attachments could be the underlying cause. Decreased or misdirected orbicularis oculi muscle function may also play a role in the lid rolling outward.

Entropic lids have more significant retractor abnormalities than the ectropic lids. In order to develop entropion, a very lax or completely disinserted retractor is necessary, which may explain why entropion is more likely to be unilateral in its presentation.

Lids with either entropion or ectropion have numerous significant abnormalities and differences compared to age-matched controls. This supports the clinical observation that surgical repair is most successful when multiple anatomical abnormalities are addressed. Entropion and ectropion repair share some common anatomic surgical considerations. The more pronounced lower lid retractor dehiscence or disinsertion found in entropion and the poor snapback characteristics in ectropion may explain why involutional entropion and ectropion are rarely seen in opposite eyes of a single patient.

## Figures and Tables

**Table 1:** Descriptive statistics for the age-matched control group, entropion group, entropion control group, ectropion group, and ectropion control group.

	Control	Entropion	Entropion control	Ectropion	Ectropion control
Number of patients	26	26	22	19	7
Number of eyes	52	30	22	30	7
Mean (years)	75	76	76	81	84
Median (years)	77	78	78	81	83
Standard deviation	10	9	9	8	10
Range (years)	56–89	53–85	53–85	57–92	75–92
Number of males	13	13	10	12	2
Number of eyes	26	16	10	21	2
Mean (years)	71	72	74	79	82
Median (years)	70	74	78	81	82
Standard deviation	9	10	10	8	10
Range (years)	59–89	53–85	53–84	57–89	75–89
Number of females	13	13	12	7	5
Number of eyes	26	14	12	9	5
Mean (years)	79	79	78	84	82
Median (years)	82	81	79	82	83
Standard deviation	9	6	6	7	6
Range (years)	56–87	70–85	70–85	75–92	80–92

**Table 2:** Descriptive statistics for each clinical measurement.

Clinical measurement	Data type	Control	Entropion	Entropion control	Ectropion	Ectropion control	Statistical test
MRD_2_ (mm)	Mean	4.8	5.0	4.9	5.1	4.6	IS *t*-test
	Stand. dev.	0.6	1.4	1.0	1.5	1.2	
	Median	5.0	5.0	5.0	5.0	5.0	
	Range	3.0–6.0	2.5–7.0	2.0–7.0	2.0–8.0	2.0–5.5	

Lid crease height (mm)	Mean	3.5	4.5	3.2	3.9	3.2	IS *t*-test
	Stand. dev.	0.5	1.2	0.6	2.0	0.6	
	Median	3.5	4.8	3.0	4.0	5.0	
	Range	2.5–4.0	2.0–6.0	2.0–4.5	2.0–9.0	2.0–7.0	

Retractor dehiscence	Present	3	28	6	15	1	Fisher's
	Absent	49	2	16	15	6	

White line	Present	0	17	1	11	0	Fisher's
	Absent	52	13	21	19	7	

Orbital fat	Present	1	24	9	24	5	Fisher's
	Absent	51	6	13	6	2	

Lid excursion (mm)	Mean	5.5	3.6	5.1	3.9	4.2	IS *t*-test
	Stand. dev.	1.0	1.6	0.9	1.0	0.9	
	Median	5.0	3.5	5.0	4.0	4.5	
	Range	4.0–8.0	0.0–6.0	3.0–6.0	2.0–6.0	3.0–5.0	

Lateral canthal excursion (mm)	Mean	5.2	3.3	4.9	4.7	3.9	IS *t*-test
	Stand. dev.	0.6	1.3	0.7	0.7	1.5	
	Median	5.0	3.0	5.0	5.0	5.0	
	Range	4.0–6.0	0.0–5.0	3.0–6.0	2.0–7.0	1.0–5.0	

Snapback test	Mean	2.0	2.4	2.1	2.8	2.9	M-W-*U*
	Stand. dev.	0.7	0.9	0.8	0.6	0.4	
	Median	2.0	3.0	2.0	3.0	3.0	

Lid distraction (mm)	Mean	7.4	9.0	9.0	10.1	9.0	IS *t*-test
	Stand. dev.	1.6	2.3	2.3	1.6	1.3	
	Median	7.0	9.0	9.0	10	8	
	Range	5.0–10.0	5.0–16.0	5.0–16.0	7.0–16.0	8.0–11.0	

IS *t*-test: independent samples *t*-test. Fisher's: Fisher's exact test. M-W-*U*: Mann-Whitney *U* test. Stand. dev.: standard deviation. (mm): millimeters.

Values are per eye, not per patient.

**Table 3:** Comparison analysis for all groups.

Clinical measurement	Control versus entropion	Control versus ectropion	Entropion versus ectropion	Entropion versus entropion control	Ectropion versus ectropion control	Control versus entropion control	Control versus ectropion control
MRD_2_	*P* = 0.324 CI = −0.6358–0.2127	*P* = 0.210 CI = −0.7588–0.1691	*P* = 0.823 CI = −08.24–0.6587	*P* = 0.693 CI = −0.5522–0.8250	*P* = 0.413 CI = −0.7418–1.7656	*P* = 0.684 CI = −0.4424–0.2920	*P* = 0.423 CI = −0.3210–0.7551

Lid crease height	*P* = 0.000 CI = −1.3923–−0.6099	*P* = 0.163 CI = −0.9950–1.707	*P* = 0.269 CI = −0.4695–1.6473	*P* = 0.000 CI = 0.6460–1.8485	*P* = 0.252 CI = −2.6701–0.7725	*P* = 0.065 CI = −0.0158–0.5081	*P* = 0.000 CI = −1.9763–−0.7956

Retractor dehiscence	*P* = 0.000	*P* = 0.000	*P* = 0.000	*P* = 0.000	*P* = 0.113	*P* = 0.017	*P* = 0.405

White line	*P* = 0.000	*P* = 0.000	*P* = 0.195	*P* = 0.000	*P* = 0.0797	*P* = 0.297	*P* = 1.000

Orbital fat	*P* = 0.000	*P* = 0.000	*P* = 1.000	*P* = 0.008	*P* = 0.631	*P* = 0.000	*P* = 0.000

Lid excursion	*P* = 0.000 CI = 1.3141–2.4231	*P* = 0.000 CI = 1.1019–2.0020	*P* = 0.361 CI = −1.0046–0.3712	*P* = 0.000 CI = −2.2804–−0.7802	*P* = 0.466 CI = −1.1801–0.5515	*P* = 0.161 CI = −0.1382–0.8148	*P* = 0.002 CI = 0.4709–2.0044

Lateral canthal excursion	*P* = 0.000 CI = 1.2083–0.4820	*P* = 0.126 CI = −0.1052–0.8347	*P* = 0.001 CI = −2.007–−0.5589	*P* = 0.000 CI = −2.1370–−0.8812	*P* = 0.199 CI = −0.4548–2.1072	*P* = 0.422 CI = −0.2040–0.4820	*P* = 0.001 CI = 0.5199–1.8619

Snapback test	*U* = 548.5 *Z* = −2.386 *P* = 0.017	*U* = 286.0 *Z* = −5.161 *P* = 0.000	*U* = 328.0 *Z* = −2.327 *P* = 0.020	*U* = 271.0 *Z* = −1.190 *P* = 0.234	*U* = 105.0 *Z* = 0.00 *P* = 1.000	*U* = 503.5 *Z* = −0.886 *P* = 0.376	*U* = 58.5 *Z* = −3.165 *P* = 0.002

Lid distraction	*P* = 0.000 CI = −3.0460–−1.3917	*P* = 0.000 CI = −3.4398–−1.8909	*P* = 0.738 CI = −0.4466–0.4997	*P* = 0.375 CI = −0.6933–1.8049	*P* = 0.154 CI = −0.4131–2.5131	*P* = 0.001 CI = −2.6231–−0.7029	*P* = 0.015 CI = −2.9114–−0.3194

*P*: the statistical value for the independent samples *t*-test or Fisher's exact test for a 95% confidence level. CI: confidence interval. *U*: test statistic. *Z*: test statistic reported for the Mann-Whitney *U* test that indicates if the two samples being compared come from the same underlying distribution at the *P* = 0.05 significance level. A *Z* score of less than 1.96 indicates that the two samples come from the same underlying distribution.

**Table 4:** Summary of statistically significant findings.

Clinical measurement	Control versus entropion	Control versus ectropion	Entropion versus ectropion	Entropion versus entropion opposite lid	Ectropion versus ectropion opposite lid	Control versus entropion opposite lid	Control versus ectropion opposite lid
MRD_2_	−	−	−	−	−	−	−
Lid crease height	+	−	−	+	−	−	+
Retractor dehiscence	+	+	+	+	−	+	−
White line	+	+	−	+	−	−	−
Orbital fat	+	+	−	+	−	+	+
Lid excursion	+	+	−	+	−	−	+
Lateral canthal excursion	+	−	+	+	−	−	+
Snapback test	+	+	+	−	−	−	+
Lid distraction	+	+	−	−	−	+	+

“+” indicates that there was a statistically significant difference between the two groups with a *P* value of less than or equal to 0.05. “−” indicates that there was no statistically significant difference between the two groups with a *P* value of greater than 0.05.
